# Demographic Factors and Academic Outcomes Associated With Taking a Leave of Absence From Medical School

**DOI:** 10.1001/jamanetworkopen.2020.33570

**Published:** 2021-01-22

**Authors:** Mytien Nguyen, Seo Ho Song, Angelina Ferritto, Ashar Ata, Hyacinth R. C. Mason

**Affiliations:** 1 School of Medicine, Yale University, New Haven, Connecticut; 2 Geisel School of Medicine, Dartmouth College, Hanover, New Hampshire; 3 College of Arts and Sciences, Emory University, Atlanta, Georgia; 4 Albany Medical College, Albany, New York

## Abstract

This cross-sectional study uses 2007-2012 data from the Association of American Medical Colleges’ Matriculating Student Questionnaire to evaluate student factors and academic outcomes associated with taking a leave of absence from medical school.

## Introduction

To address the US physician shortage, the likelihood that medical matriculants transition to graduation and physicianhood must increase.^1^ One factor that has negative consequences for medical school graduation rates is taking a leave of absence (LOA).^2^ Students take LOAs for academic, financial, health, or personal reasons. This study of US medical school matriculants explores potential factors and academic outcomes associated with taking an LOA.

## Methods

This cross-sectional study was reviewed and approved by the Albany Medical College institutional review board, which waived the need for informed consent because the data were from a national data set and were deidentified. The study followed the Strengthening the Reporting of Observational Studies in Epidemiology (STROBE) reporting guideline. We used deidentified, individual-level data provided by the Association of American Medical Colleges (AAMC)^3,4,5^ for 48 406 medical matriculants from academic years 2007-2008 to 2011-2012. The AAMC Matriculating Student Questionnaire represents between 68.2% and 78.4% of matriculating students and includes complete data for fields of interest. It follows participants for 5 years after matriculation. The majority of medical students pursuing only a doctor of medicine graduate in 5 years.^1^

Premedical school experiences associated with the likelihood of an LOA were examined using a multivariable logistic regression model. Factors associated with an LOA included Medical College Admission Test (MCAT) scores, being a first-generation (no parent with a 4-year degree) or continuing-generation (at least 1 parent with a 4-year degree) college graduate, sex, racial/ethnic identity, matriculation age, the Carnegie classification of the student’s undergraduate institution, premedical school experiences, and parental household income. The MCAT metrics were for the MCAT version used from 1991 through January 2015. The composite score ranges from 3 to 45, with lower scores indicating fewer correct answers. The first-generation variable was derived from the American Medical College Application Service; applicants who responded “some college or less” for both parents or guardians were included in the first-generation classification. Race/ethnicity was derived from self-identification from students who were US citizens or permanent residents.

Statistical analyses were conducted from July 8, 2020, to August 15, 2020, using Stata, version 16.1 (StataCorp LLC). We report odds ratios (ORs) and 95% CIs for each logistic regression model. Statistical significance was set at *P* < .05 using a 2-tailed test.

## Results

Among the 48 406 participants in the study, 28 994 (59.9%) were from households within the highest 2 income quintiles, 23 740 (49.0%) were men, and 31 176 (64.4%) identified as non-Hispanic White individuals. A total of 2786 students took an LOA. Students who took an LOA were more likely to withdraw or be dismissed from medical school. In addition, graduation was inversely associated with likelihood of taking an LOA (OR, 0.74; 95% CI, 0.70-0.78) (Table 1).

**Table 1.** Academic Outcomes of Matriculating Student Questionnaire Participants Who Matriculated Between 2007-2008 and 2011-2012 Who Did and Did Not Take a Leave of Absence During Medical School

Leave of absence	Graduates (n = 48 406)		Dismissals and withdrawals			
			Academic (n = 550)		Nonacademic (n = 724)	
	No. (%)	OR (95% CI)	No. (%)	OR (95% CI)	No. (%)	OR (95% CI)
No	45 620 (94.24)	1 [Reference]	253 (46.00)	1 [Reference]	228 (31.49)	1 [Reference]
Yes	2786 (5.76)	0.74 (0.70-0.78)	297 (54.00)	13.95 (11.74-16.57)	496 (68.51)	35.62 (30.31-41.86)

Abbreviation: OR, odds ratio.

Students who were more likely to take an LOA were from households with lower (vs higher) income (adjusted OR, 1.19; 95% CI, 1.09-1.31), non-White racial/ethnic groups (non-Hispanic Black or African American: adjusted OR, 1.34 [95% CI, 1.13-1.59]; non-Hispanic Asian, Native Hawaiian, or Pacific Islander: adjusted OR, 1.37 [95% CI, 1.22-1.54]; non-Hispanic American Indian or Alaska Native: adjusted OR, 2.02 [95% CI, 1.29-3.19]; non-Hispanic multiracial or other: adjusted OR, 1.81 [95% CI, 1.58-2.09]; and Hispanic or Latinx: adjusted OR, 1.30 [95% CI, 1.09-1.56]), as well as participants in college summer enrichment (adjusted OR, 1.16; 95% CI, 1.03-1.31) and postbaccalaureate premedical school programs (adjusted OR, 1.26; 1.10-1.45) (Table 2). Students with higher MCAT scores (adjusted OR, 0.95; 95% CI, 0.94-0.96) and college laboratory or health volunteer or work experiences (adjusted OR, 0.84; 95% CI, 0.72-0.99) were less likely to take an LOA.

**Table 2.** Multivariate Logistic Regression of Taking a Leave of Absence by Medical Students’ Demographic Characteristics and Premedical Experiences Among MSQ Participants Who Matriculated From 2007-2008 Through 2011-2012

Characteristic	MSQ participants, No. (%)			AOR (95% CI)
	All	Leave of absence		
		No	Yes	
Total	48 406 (100)	45 620 (94.2)	2786 (5.8)	NA
First-generation college graduate				
No	42 351 (87.5)	39 995 (87.7)	2356 (84.6)	1 [Reference]
Yes	6055 (12.5)	5625 (12.3)	430 (15.4)	0.97 (0.86-1.10)
Parental household income, quintiles^a^				
Top 2	28 994 (59.9)	27 517 (60.3)	1477 (53.0)	1 [Reference]
Bottom 3	13 648 (28.2)	12 687 (27.8)	961 (34.5)	1.19 (1.09-1.31)
Premedical loan				
No	29 646 (61.2)	28 014 (61.4)	1632 (58.6)	1 [Reference]
Yes	17 714 (36.6)	16 622 (36.4)	1092 (39.2)	0.94 (0.87-1.04)
Race/ethnicity				
Non-Hispanic				
White	31 176 (64.4)	29 685 (65.1)	1491 (53.5)	1 [Reference]
Black or African American	2489 (5.1)	2258 (4.9)	231 (8.3)	1.34 (1.13-1.59)
Asian, Native Hawaiian, or other Pacific Islander	8456 (17.5)	7921 (17.4)	535 (19.2)	1.37 (1.22-1.54)
American Indian or Alaska Native alone	204 (0.4)	181 (0.4)	23 (0.8)	2.02 (1.29-3.19)
Hispanic or Latinx	2775 (5.7)	2609(5.7)	166 (6.0)	1.30 (1.09-1.56)
Non-Hispanic multiracial, other, or unknown^b^	3306 (6.8)	2966 (6.5)	340 (12.2)	1.81 (1.58-2.09)
Sex				
Male	23 740 (49.0)	22 350 (49.0)	1390 (49.9)	1 [Reference]
Female	24 666 (51.0)	23 270 (51.0)	1396 (50.1)	1.03 (0.95-1.13)
Age at matriculation, y				
<23	20 317 (42.0)	19 331 (42.4)	986 (35.4)	1 [Reference]
≥23	28 089 (58.0)	26 289 (57.6)	1800 (64.6)	1.17 (1.07-1.29)
MCAT score, mean (SD)	29.87 (4.89)	29.97 (4.83)	28.26 (5.48)	0.94 (0.94-0.96)
Carnegie classification of undergraduate degree-granting institution				
Research universities, very high research activity	30 305 (62.6)	28 607 (62.7)	1698 (60.9)	1 [Reference]
Baccalaureate colleges, arts and sciences	6394 (13.2)	6062 (13.3)	332 (11.9)	0.93 (0.82-1.06)
Master’s colleges and universities	5105 (10.5)	4766 (10.4)	339 (12.2)	1.04 (0.91-1.19)
Research universities and doctoral research universities, high research activity	6602 (13.6)	6185 (13.6)	417 (15.0)	0.97 (0.86-1.10)
Participation in magnet high school				
No	45 316 (93.6)	42 749 (93.7)	2567 (92.1)	1 [Reference]
Yes	3090 (6.4)	2871 (6.3)	219 (7.9)	1.13 (0.97-1.33)
Participation in high school research laboratory				
No	43 443 (89.7)	40 954 (89.8)	2489 (89.3)	1 [Reference]
Yes	4963 (10.3)	4666 (10.2)	297 (10.7)	1.04 (0.91-1.20)
Participation in college research laboratory				
No	21 046 (43.5)	19 728 (43.2)	1318 (47.3)	1 [Reference]
Yes	27 360 (56.5)	25 892 (56.8)	1468 (52.7)	0.90 (0.83-0.98)
Participation in college summer program				
No	42 682 (88.2)	40 317 (88.4)	2365 (84.9)	1 [Reference]
Yes	5724 (11.8)	5303 (11.6)	421 (15.1)	1.16 (1.03-1.31)
Participation in MCAT preparation course				
No	14 673 (30.3)	13 880 (30.4)	793 (28.5)	1 [Reference]
Yes	33 733 (69.7)	31 740 (69.6)	1993 (71.5)	1.02 (0.93-1.12)
Participation in postbaccalaureate premedical program				
No	44 160 (91.2)	41 689 (91.4)	2471 (88.7)	1 [Reference]
Yes	4246 (8.8)	3931 (8.6)	315 (11.3)	1.26 (1.10-1.45)
Volunteered or worked in health care field				
No	3045 (6.3)	2826 (6.2)	219 (7.9)	1 [Reference]
Yes	45 361 (93.7)	42 794 (93.8)	2567 (92.1)	0.84 (0.72-0.99)

Abbreviations: AOR, adjusted odds ratio; MCAT, Medical College Admission Test; MSQ, Matriculating Student Questionnaire; NA, not applicable.

^a^ Annual household income in the top 2 quintiles was greater than or equal to $80 080. In the bottom 3 quintiles, annual household income was less than $80 080.

^b^ The non-Hispanic multiracial, other, or unknown category included respondents who indicated that they self-identified with more than one racial/ethnic category or who chose other from the list of racial/ethnic categories on the survey.

## Discussion

The findings of this cross-sectional study suggest that medical students who took an LOA were less likely to graduate and more likely to withdraw or be dismissed from medical school than their peers who did not take an LOA. Students who identified as non-White individuals or were from households with incomes within the lower 3 quintiles were more likely than peers in the upper quintiles to take an LOA. Although racial/ethnic disparities in medical graduation rates have been shown,^2,6^ to our knowledge, this study is the first to examine racial/ethnic and income disparities between students who take an LOA from medical school and those who do not. This study has limitations. Causation cannot be inferred owing to the observational cross-sectional nature of the study, and variables in this study were self-reported, thereby introducing potential self-protection bias. Future research should be aimed at understanding the complex factors associated with the need to take an LOA and reasons why students take an LOA. Such research is needed to inform tailored interventions that identify students who are more likely to take an LOA to increase the likelihood that all students, including those who take an LOA, thrive in medical school and become the future physicians they aspire to be.
